# Age-dependent variations in haematological and serum biochemical parameters of domestic pigeons (*Columba livia domestica*)

**DOI:** 10.1016/j.heliyon.2021.e07486

**Published:** 2021-07-05

**Authors:** Ochuko Orakpoghenor, Talatu Patience Markus, Ngozi Ejum Ogbuagu, Samson James Enam, Sunday Blessing Oladele, Paul Ayuba Abdu, King Akpofure Nelson Esievo

**Affiliations:** a Veterinary Pathology, Ahmadu Bello University, Zaria, Nigeria; b Veterinary Microbiology, Ahmadu Bello University, Zaria, Nigeria; c Veterinary Physiology, Ahmadu Bello University, Zaria; d Veterinary Medicine, Ahmadu Bello University, Zaria, Nigeria

**Keywords:** Age-dependent, Domestic pigeons, Blood, Serum, Variation

## Abstract

In this study, the age-dependent variations in haematological and serum biochemical parameters of domestic pigeons were evaluated. Sixty apparently healthy domestic pigeons comprising 30 young (2–7 weeks of age) and 30 adult (>7 weeks of age) were sampled from local breeders. Blood was collected from each bird via brachial venipuncture and divided into 2 parts; one part dispensed into labeled tubes containing ethylenediaminetetraacetic acid as anticoagulant was processed for haematological analyses. The other part was dispensed into labeled plain tubes, serum harvested and processed for serum biochemical analyses. Results revealed overall packed cells volume (PCV), haemoglobin concentration (HGB) and red blood cells (RBC) of 42.97 ± 4.53%, 13.15 ± 1.82 g/dL and 3.63 ± 0.50 × 10^12^/L respectively. All haematological parameters except mean corpuscular haemoglobin (MCH), mean corpuscular haemoglobin concentration (MCHC) and lymphocyte count showed statistical (p < 0.05) differences between young and adult pigeons. Values recorded for serum total protein, albumin, globulin, serum/albumin ratio, urea, creatinine and urea/creatinine ratio were 4.32 ± 0.74 g/dL, 2.07 ± 0.30 g/dL, 2.25 ± 0.74 g/dL, 1.04 ± 0.43, 0.48 ± 0.33 mg/dL, 0.75 ± 0.52 mg/dL and 0.73 ± 0.51 respectively. Serum urea and creatinine concentrations were significantly (p < 0.05) higher in adult (0.62 ± 0.40; 1.04 ± 0.60 mg/dL) compared to young (0.34 ± 0.13; 0.47 ± 0.15 mg/dL) pigeons. This study therefore demonstrated age-dependent variations in haematological and serum biochemical parameters of domestic pigeons.

## 1 Introduction

Domestic pigeons (*Columba livia*) are subspecies of the rock pigeon that is believed to come into existence following prolonged domestication (Blechman, 2007). Domestic pigeons tend to live amongst humans and other animals with history of their considerable contribution in terms of war where they serve as messengers (Levi 1974; Wiltschko and Wiltschko 2003). They have been documented to be extremely protective of their eggs, with reports of them attacking strangers' interference on their productive process (Lack 2003; Shapiro and Domyan 2013).

Domestic pigeons are bred as sources of meat, hobby, symbol (peace, love, purity and innocence), manure, feather products and for experimental purposes (Fakhri et al., 2013; Ihedioha et al., 2016). The adaptations of domestic pigeons to life in the city have led to their abundance in urban areas and dependence upon humans resulting from conscious feeding by bird lovers (Bahrami et al., 2013). Due to their interaction with man and other animals, pigeons have been proposed as potential carriers of zoonotic disease causing agents. Due to the increase in the consumption of pigeons, there is need for assessment of markers that would be valuable in terms of production. These markers include haematological and biochemical profiles as the blood constitutes the major means by which several substances are being transported in the body (Garraud and Tissot 2018). This is important because changes in blood parameters could depict deviations from the normal and this could be caused by metabolic distortion, pathogen invasion, deprivation, stress and other forms of injury/insult (Ihedioha et al., 2012). However in avian species, haematological assessment has been reported to be a suitable tool in ascertaining health and nutrition status, disease diagnosis, prognosis and efficacy of therapy (Campbell, 1998; Clark et al., 2009; Ihedioha et al., 2011). Hence in this study, the age-dependent variations in haematological and serum biochemical parameters of domestic pigeons were evaluated to propel researches using these birds as experimental models.

## 2 Materials and methods

### 2.1 Experimental animals

A total of sixty domestic pigeons (*Columba livia*) comprising 30 each of young (2–7 weeks of age) and adult (>7 weeks of age) were sampled in this study. The pigeons were obtained from local breeders in households and live bird markets. Before sampling, the birds were physically examined for evidence of diseases and/or abnormalities. Information on the ages of the birds was obtained from the breeders. The sampling method was purposive based on convenience, availability and willingness of the breeders to allow sampling of the birds.

### 2.2 Blood collection

Blood (2 mL) was collected from each pigeon by brachial venipuncture and divided into 2 parts. One part was dispensed in labeled sample tube containing ethylene diamine tetraacetic acid (EDTA) as anticoagulant and processed for haematological analyses. The other part was dispensed in plain sample tube, serum was harvested and processed for serum biochemical analyses.

### 2.3 Haematological analyses

Packed cells volume (PCV) was determined using microhaematocrit technique as described by Rehman et al. (2003). Haemoglobin concentration was assayed colorimetrically using the cyanomethhaemoglobin method (Higgins et al., 2008). Erythrocytes and total leukocyte count were determined by haemocytometer method using Improved Neubauer haemocytometer and Natt-Herrick solution (1:200 dilution) as diluting fluid (Campbell and Ellis 2007). For differential leukocyte count, smears were prepared, stained using Giemsa technique and cells counted by battlement counting method (Thrall and Weiser 2002). Mean corpuscular volume (MCV), mean corpuscular haemoglobin (MCH) and mean corpuscular haemoglobin concentration (MCHC) were calculated using standard formula outlined by Campbell (1994).

### 2.4 Serum biochemical analyses

Serum total protein, albumin, urea and creatinine concentrations were determined by semi-autoanalyzer technique following the manufacturer's procedures (Agappe Diagnostics Switzerland GmbH). Serum globulin concentration was determined using standard formula by subtracting albumin concentration from total protein concentration. Albumin/globulin and urea/creatinine ratios were calculated using standard formula (Lumeij 2008).

### 2.5 Ethical approval

The use of animals in this study was approved by the Ahmadu Bello University Committee on Animal Use and Care (ABUCAUC).

### 2.6 Data analyses

Data were presented in tables as mean ± standard deviation with range (minimum and maximum values). The data were analyzed using Statistical Package for Social Sciences, SPSS and independent Students' t-test to test for differences between ages of birds in all the parameters. Probability values of p ≤ 0.05 were considered significant.

## 3 Results

### 3.1 Haematology

Overall packed cells volume (PCV), haemoglobin concentration (HGB) and red blood cells (RBC) of 42.97 ± 4.53%, 13.15 ± 1.82 g/dL and 3.63 ± 0.50 × 10^12^/L respectively were recorded (Table 1). Packed cells volume (PCV), haemoglobin concentration (HGB) and erythrocytes count (RBC) were significantly (p < 0.05) higher in adult (45.70 ± 4.09%; 14.18 ± 1.47 g/dL; 3.98 ± 0.32 × 10^12^/L) compared to young (40.23 ± 3.10%; 12.11 ± 1.53 g/dL; 3.29 ± 0.39 × 10^12^/L) pigeons (Table 1).

**Table 1:** Haematological and serum biochemical parameters of domestic pigeons.

Parameter	Mean ± SD	Range	95 % CI
**Erythrocytic parameters**			
Packed cells volume (%)	42.97 ± 4.53	32.0–53.0	41.8–44.14
Haemoglobin concentration (g/dL)	13.15 ± 1.82	9.46–16.46	12.68–13.61
Red blood cells (× 10^12^/L)	3.63 ± 0.50	2.73–4.53	3.51–3.76
Mean corpuscular volume (*fL*)	120.9 ± 13.41	90.23–150.5	117.5–124.4
Mean corpuscular haemoglobin (pg)	36.49 ± 5.11	21.91–51.67	35.17–37.81
Mean corpuscular haemoglobin concentration (g/dL)	30.28 ± 3.70	18.71–40.33	29.33–31.24
**Leukocytic parameters**			
Total leukocyte count (× 10^9^/L)	23.35 ± 3.60	16.2–19.9	22.42–24.28
Heterophils count (× 10^9^/L)	8.62 ± 1.84	5.6–14.66	8.14–9.09
Lymphocytes count (× 10^9^/L)	13.12 ± 1.81	8.5–18.73	12.66–13.59
Heterophil/lymphocyte ratio	0.64 ± 0.18	0.43–1.18	0.59–0.69
Monocytes count (× 10^9^/L)	1.41 ± 1.00	0.4–4.83	1.15–1.66
Eosinophils count (× 10^9^/L)	0.32 ± 0.31	0.0–1.02	0.24–0.40
Basophils count (× 10^9^/L)	0.14 ± 0.20	0.0–0.79	0.09–0.19
**Serum biochemical parameters**			
Total protein (g/dL)	4.32 ± 0.74	3.2–6.2	4.13–4.51
Albumin (g/dL)	2.07 ± 0.30	1.52–2.73	1.99–2.15
Globulin (g/dL)	2.25 ± 0.74	1.03–4.25	2.05–2.44
Albumin/globulin ratio	1.04 ± 0.43	0.39–2.40	0.93–1.15
Urea (mg/dL)	0.48 ± 0.33	0.16–1.57	0.39–0.56
Creatinine (mg/dL)	0.75 ± 0.52	0.24–2.76	0.62–0.88
Urea/creatinine ratio	0.73 ± 0.51	0.10–3.86	0.60–0.86

The values calculated for mean corpuscular volume (MCH), mean corpuscular haemoglobin (MCH) and mean corpuscular haemoglobin concentration (MCHC) were 120.9 ± 13.41 fL, 36.49 ± 5.11 pg and 30.28 ± 3.70 g/dL respectively (Table 1). Mean corpuscular volume (MCV) showed statistical (p < 0.05) difference between young (126.7 ± 13.21 fL) and adult (115.20 ± 11.10 fL) pigeons. There was no statistical (p > 0.05) difference for MCH and MCHC between young (37.21 ± 6.00 pg; 29.34 ± 3.43 g/dL) and adult (35.77 ± 4.02 pg; 31.22 ± 3.77 g/dL) pigeons (Table 2).

**Table 2:** Erythrocytic parameters of young and adult domestic pigeons.

Parameter	Mean ± SD		Range	
	2–7 weeks	>7 weeks	2–7 weeks	>7 weeks
Packed cells volume (%)	40.23 ± 3.10^a^	45.70 ± 4.09^b^	32.00–45.00	36.0–53.0
Haemoglobin concentration (g/dL)	12.11 ± 1.53^a^	14.18 ± 1.47^b^	9.46–14.76	9.73–16.45
Erythrocyte count (× 10^12^/L)	3.29 ± 0.39^a^	3.98 ± 0.32^b^	2.73–4.19	2.99–4.53
Mean corpuscular volume (*fL*)	126.7 ± 13.21^a^	115.20 ± 11.10^b^	99.75–150.2	90.23–150.5
Mean corpuscular haemoglobin (pg)	37.21 ± 6.00^a^	35.77 ± 4.02^a^	24.61–51.67	21.91–48.56
Mean corpuscular haemoglobin concentration (g/dL)	29.34 ± 3.43^a^	31.22 ± 3.77^a^	22.52–37.36	18.71–40.33

Values with different superscript alphabet for the same parameter differ significantly at p < 0.05.

Leukocytic parameters recorded were total leukocytes count (23.35 ± 3.60 × 10^9^/L), heterophils (8.62 ± 1.84 × 10^9^/L), lymphocytes (13.12 ± 1.81 × 10^9^/L), heterophil/lymphocyte ratio (0.64 ± 0.18), monocytes (1.41 ± 1.00 × 10^9^/L), eosinophils (0.32 ± 0.31 × 10^9^/L) and basophils (0.14 ± 0.20 × 10^9^/L) (Table 1). Total leukocytes count (TLC) was significantly (p < 0.05) higher in adult (25.32 ± 3.35 × 10^9^/L) compared to young (21.38 ± 2.67 × 10^9^/L) pigeons (Table 2). No statistical difference existed for lymphocyte count between young (13.16 ± 1.48 × 10^9^/L) and adult (13.09 ± 2.12 × 10^9^/L) pigeons. Significantly (p < 0.05) higher values were recorded in adult compared to young pigeons for heterophils (9.55 ± 1.89 × 10^9^/L; 7.68 ± 1.22 × 10^9^/L), monocytes (2.03 ± 1.08 × 10^9^/L; 0.78 ± 0.26 × 10^9^/L), eosinophil (0.45 ± 0.33 × 10^9^/L; 0.19 ± 0.21 × 10^9^/L), basophil (0.21 ± 0.25 × 10^9^/L; 0.07 ± 0.11 × 10^9^/L) counts and heterophil/lymphocyte (H/L) ratio (0.75 ± 0.20 × 10^9^/L; 0.53 ± 0.07 × 10^9^/L) (Table 3).

**Table 3:** Leukocytic parameters of young and adult domestic pigeons.

Parameter	Mean ± SD		Range	
	2–7 weeks	>7 weeks	2–7 weeks	>7 weeks
Total leukocyte count (× 10^9^/L)	21.38 ± 2.67^a^	25.32 ± 3.35^b^	16.20–25.90	19.90–32.30
Heterophil (× 10^9^/L)	7.68 ± 1.22^a^	9.55 ± 1.89^b^	5.60–10.60	6.85–14.66
Lymphocyte (× 10^9^/L)	13.16 ± 1.48^a^	13.09 ± 2.12^a^	10.80–16.60	8.50–18.73
Heterophil/lymphocyte ratio	0.53 ± 0.07^a^	0.75 ± 0.20^b^	0.43–0.68	0.46–1.18
Monocyte (× 10^9^/L)	0.78 ± 0.26^a^	2.03 ± 1.08^b^	0.40–1.20	0.66–4.80
Eosinophil (× 10^9^/L)	0.19 ± 0.21^a^	0.45 ± 0.33^b^	0.00–0.80	0.00–1.02
Basophil (× 10^9^/L)	0.07 ± 0.11^a^	0.21 ± 0.25^b^	0.00–0.25	0.00–0.79

Values with different superscript alphabet for the same parameter differ significantly at p < 0.05.

### 3.2 Serum biochemistry

Values recorded for serum total protein, albumin, globulin, serum/albumin ratio, urea, creatinine and urea/creatinine ratio were 4.32 ± 0.74 g/dL, 2.07 ± 0.30 g/dL, 2.25 ± 0.74 g/dL, 1.04 ± 0.43, 0.48 ± 0.33 mg/dL, 0.75 ± 0.52 mg/dL and 0.73 ± 0.51 respectively (Table 1). Serum total protein, albumin and globulin concentrations determined for young pigeons were 4.20 ± 0.74 g/dL, 2.06 ± 0.32 g/dL and 2.12 ± 0.72 g/dL respectively, and these showed no statistical (p > 0.05) difference when compared to those of adult pigeons with values of 4.44 ± 0.74 g/dL, 2.08 ± 0.29 g/dL and 2.37 ± 0.76 g/dL respectively (Table 4).

**Table 4:** Serum biochemical parameters of young and adult domestic pigeons.

Parameter	Mean ± SD		Range	
	2–7 weeks	>7 weeks	2–7 weeks	>7 weeks
Total protein (g/dL)	4.20 ± 0.74^a^	4.44 ± 0.74^a^	3.30–5.80	3.20–6.20
Albumin (g/dL)	2.06 ± 0.32^a^	2.08 ± 0.29^a^	1.52–2.73	1.57–2.72
Globulin (g/dL)	2.12 ± 0.72^a^	2.37 ± 0.76^a^	1.09–3.67	1.03–4.25
Albumin/globulin ratio	1.09 ± 0.41^a^	1.00 ± 0.45^a^	0.53–1.99	0.39–2.40
Urea (mg/dL)	0.34 ± 0.13^a^	0.62 ± 0.40^b^	0.16–0.68	0.17–1.57
Creatinine (mg/dL)	0.47 ± 0.15^a^	1.04 ± 0.60^b^	0.24–0.82	0.29–2.76
Urea/creatinine ratio	0.69 ± 0.05^a^	0.77 ± 0.73^a^	0.49–0.81	0.10–3.86

Values with different superscript alphabet for the same parameter differ significantly at p < 0.05.

Serum urea and creatinine concentrations were significantly (p < 0.05) higher in adult (0.62 ± 0.40 mg/dL; 1.04 ± 0.60 mg/dL) compared to young (0.34 ± 0.13 mg/dL; 0.47 ± 0.15 mg/dL) pigeons. No statistical (p > 0.05) difference existed for albumin/globulin and urea/creatinine ratios of young (1.09 ± 0.41; 0.69 ± 0.05) and adult (1.00 ± 0.45; 0.77 ± 0.73) pigeons (Table 4).

## 4 Discussion

In this study, haematological and serum biochemical parameters of apparently healthy domestic pigeons in Zaria and its environs were evaluated. The overall PCV recorded (42.97 ± 4.53%) was similar to the 42.5 % reported by Ritchie et al. (1994) but lower than 44.54 ± 4.73% reported by Ihedioha et al. (2016) for domestic pigeons and 49.36 ± 6.40% reported by Khan et al. (2011) for street rock pigeons. The HGB of pigeons (13.15 ± 1.82 g/dL) and RBC (3.63 ± 0.50 × 10^12^/L) in this study were slightly lower than the 14.46 ± 0.19 g/dL and 3.96 ± 0.05 × 10^12^/L reported by Lashev et al. (2009) but higher than 12.89 ± 1.55 g/dL and 3.34 ± 0.38 × 10^12^/L reported by Ihedioha et al. (2016). Also the erythrocytic indices (MCV, MCH and MCH) and leukocytic parameters in this study showed significant variation with those reported by Ihedioha et al. (2016) for domestic pigeons and Khan et al. (2011) for street rock pigeons. Study by Opara et al. (2012) showed comparable but slight differences in the serum biochemical parameters assayed for in this study. The disparity between observations in this study and those by other researchers may be due to differences in geographical locations, seasons, climate, feeding, and other environmental factors.

There was higher PCV, haemoglobin and RBC in adult compared to young pigeons in this study. This may be due to physiological adjustment induced by greater demands for continuous flight in adult pigeons as a requirement in search for feed (Viscor et al., 1985; Butler 2016). This is so as continuous flight movement by adult pigeons induces hypoxia leading to increase in their oxygen demand. The increased oxygen demand is compensated for by increase in oxygen carrying capacity reflected by the increase in PCV, haemoglobin and RBC as observed in adult pigeons in this study (Scott 2011; Butler 2016). In contrast, young pigeons do not move around as they are still learning the act of flying and so are being fed by the adult. Their restricted flight movement in turn does not create that increase in oxygen demand thus resulting in the lower values of PCV, haemoglobin and RBC compared to adult (Meir et al., 2019). Also, the continuous flight movements by adult pigeons result in increased metabolism with subsequent water loss leading to dehydration (Ward et al., 2002; Meir et al., 2019). This dehydration may also contribute to the significantly higher values of PCV and RBC observed. The higher MCV in young pigeons suggests larger RBC and this may be linked to physiological lower RBC values resulting from their rapid growth rate with hemodilution from plasma volume expansion, destruction of hatchling RBC and decreased production due to low erythropoietin concentrations (Brenten et al., 2016).

The significantly (p < 0.05) higher TLC reflected by higher heterophils, monocytes, eosinophils and basophils in adult pigeons is suggestive of possible exposure to infections agents, inflammatory responses and tissue destruction. Heterophils and macrophages have been reported to phagocytize tissue debris (Broom 2019) and since adult pigeons are continuously exposed to infectious agents that may cause tissue destruction, their significantly higher heterophils and monocytes compared to those of the young are unusual. Tissues such as skin, lungs and gastrointestinal tract are rich in mast cells and when destroyed, there is degranulation of mast cells leading to histamine release and subsequent attraction of eosinophils (Beghdadi et al., 2011). This may contribute to the higher eosinophils in adult compared to young pigeons as they are in continuous flight movement with persistent exposure to these tissue destruction agents.

The excretion of urea is primarily by glomerular filtration and its reabsorption rate from the tubules is totally dependent on water availability such that there is reabsorption of all filtered urea during dehydration (Weiner et al., 2015). The dehydration induced by continuous flight in adult pigeons may be the possible reason for their significantly higher urea concentration compared to young pigeons. Also, the flight movement of pigeons involves vigorous muscular activities leading to extensive destruction of muscle fibres and consequent creatinine release (Wyss and Kaddurah-Daouk 2000; Baird et al., 2012). Since these muscular activities are minimal in young pigeons, creatinine release is decreased thus may contribute to the significantly higher creatinine concentration in adult compared to young pigeons as observed in this study.

## 5 Conclusion

This study documented age-dependent variations in the haematological and some serum biochemical parameters of domestic pigeons. Therefore, further studies on disease conditions that may alter these parameters in pigeons are recommended.

## Declarations

### Author contribution statement

Ochuko Orakpoghenor, Talatu Patience Markus: Performed the experiments; Analyzed and interpreted the data; Wrote the paper.

Ngozi Ejum Ogbuagu: Performed the experiments; Analyzed and interpreted the data.

Samson James Enam: Contributed reagents, materials, analysis tools or data.

Sunday Blessing Oladele, Paul Ayuba Abdu, King Akpofure Nelson Esievo: Conceived and designed the experiments.

### Funding statement

This research did not receive any specific grant from funding agencies in the public, commercial, or not-for-profit sectors.

### Data availability statement

Data will be made available on request.

### Declaration of interests statement

The authors declare no conflict of interest.

### Additional information

No additional information is available for this paper.

## References

[bib1] Bahrami A.M., Hosseini E., Razmjo M. (2013). Important parasites in pigeons, its haematological parameters and pathology of intestine. World Appl. Sci. J..

[bib2] Baird M.F., Graham S.M., Baker J.S., Bickerstaff G.F. (2012). Creatine-kinase and exercise-related muscle damage implications for muscle performance and recovery. J. Nutr. Metab..

[bib3] Beghdadi W., Madjene L.C., Benhamou M., Charles N., Gautier G., Launay P., Blank U. (2011). Mast cells as cellular sensors in inflammation and immunity. Front. Immunol..

[bib4] Blechman A. (2007). Pigeons-The Fascinating Saga of the World's Most Revered and Reviled Bird.

[bib5] Brenten T., Morris P.J., Salt C., Raila J., Kohn B., Schweigert F.J., Zentek J. (2016). Age-associated and breed-associated variations in haematological and biochemical variables in young Labrador retriever and miniature schnauzer dogs. Veter. Record Open.

[bib6] Broom L.J. (2019). Host-microbe interactions and gut health in poultry – focus on innate response. Microorganisms.

[bib7] Butler P.J. (2016). The physiological basis of bird flight. Phil. Trans. Biol. Sci..

[bib8] Campbell T.W., Ritchie B.W., Harrison G.J., Harrison L.R. (1994). Haematology. Avian Medicine: Principles and Application.

[bib9] Campbell T.W. (1998). Avian Haematology and Cytology.

[bib10] Campbell T.W., Ellis C.K., Campbell T.W., Ellis C.K. (2007). Haematology of birds. Avian and Exotic Animal Haematology and Cytology.

[bib11] Clark P., Boardman W.S.J., Raidal S.R. (2009). Atlas of Clinical Avian Hematology.

[bib12] Fakhri K.P., Khiabani F.S., Adelzadeh P. (2013). Pigeon; the reflection of purity and peace. J. Appl. Sci. Agri..

[bib13] Garraud O., Tissot J. (2018). Blood and blood components: from similarities to differences. Front. Med..

[bib14] Higgins T., Beutler E., Doumas B.T., Burtis C.A., Ashwood E.R., Bruns D.E. (2008). Measurement of haemoglobin in blood. Pages 514 – 515. Tietz Fundamentals of Clinical Chemistry.

[bib15] Ihedioha J.I., Anyogu D.C., Chibuezeoke K.J. (2016). Haematological profile of the domestic pigeon (*Columba livia domestica*) in Nsukka agro-ecological zone, Enugu State, Nigeria. Anim. Res. Int..

[bib16] Ihedioha J.I., Okorie-Kanu C.O., Ugwu C.P. (2011). The blood picture and serum biochemistry profile of the African pied crow (*Corvus albus*). Comp. Clin. Pathol..

[bib17] Ihedioha J.I., Ugwuja J.I., Noel-Uneke O.A., Udeani I.J., Daniel-Igwe G. (2012). Reference values for the haematology profile of conventional grade outbred albino mice (*Mus musculus*). Anim. Res. Int..

[bib18] Khan B.Y.A., Ali F., Saeed M.Q., Asghar M., Iqbal F. (2011). A study on serum biochemistry and haematology profiling of blue rock pigeon (*Columba livia*) in Multan (Punjab Pakistan). Pakistan J. Zool..

[bib19] Lack P., Pervins C. (2003). Pigeons and Doves. The New Encyclopedia of Birds.

[bib20] Lashev L., Hubenov H., Nikolov Y., Lasheva V., Mihailov R. (2009). Comparison of some haematological parameters between three bird species from the Columbidae family. Vet. Arch..

[bib21] Levi W.M. (1974). The Pigeon.

[bib22] Lumeij J.T., Kaneko J.J., Harvey J.W., Bruss M.L. (2008). Avian clinical biochmeistry. Clinical Biochemsitry of Domestic Animals.

[bib23] Meir J.U., York J.M., Chua B., Jardine W., Hawkes L.A., Milsom W.K. (2019). Reduced metabolism supports hypoxic flight in the high-flying bar-headed goose (*Anser indicus*). eLife.

[bib24] Opara M.N., Ogbuewu I.P., Iwuji C.T., Ihesie E.K., Etuk I.F. (2012). Blood characteristics, microbial and gastrointestinal parasites of street pigeons (*Columbia livia*) in Owerri, Imo State, Nigeria. Sci. J. Anim. Sci..

[bib25] Rehman H., Abbas S., Lohahet N. (2003).

[bib26] Ritchie B.W., Harrison G.J., Harrison L.R. (1994). Avian Medicine: Principles and Application.

[bib27] Scott G.R. (2011). Elevated performance: the unique physiology of birds that fly at high altitudes. J. Exp. Biol..

[bib28] Shapiro M.D., Domyan E.T. (2013). Domestic pigeons. Curr. Biol..

[bib29] Thrall M.A., Weiser M.G., Hendrix C.M. (2002). Haematology. Laboratory Procedures for Veterinary Technicians.

[bib30] Viscor G., Marques M.S., Palomeque J. (1985). Cardiovascular and body organ weight adaptations as related to flight activity in birds. Comp. Biochem. Physiol. A: Comp. Physiol..

[bib31] Ward S., Bishop C.M., Woakes A.J., Butler P.J. (2002). Heart rate and the rate of oxygen consumption of flying and walking barnacle geese (*Branta leucopsis*) and bar-headed geese (*Anser indicus*). J. Exp. Biol..

[bib32] Weiner I.D., Mitch W.E., Sands J.M. (2015). Urea and ammonia metabolism and the control of renal nitrogen excretion. Clin. J. Am. Soc. Nephrol..

[bib33] Wiltschko W., Wiltschko R. (2003). Avian navigation: from historical to modern concepts. Anim. Behav..

[bib34] Wyss M., Kaddurah-Daouk R. (2000). Creatine and creatinine metabolism. Physiol. Rev..

